# CRISPR-Cas9 editing of *TNFAIP3* variants in salivary gland epithelial cells to study Sjögren’s disease pathogenesis

**DOI:** 10.3389/fgeed.2025.1625393

**Published:** 2025-07-23

**Authors:** Subhashis Ghosh, Qisheng Tu, Zoe Xiaofang Zhu, Sreelakshmi Panginikkod, Jake Jinkun Chen

**Affiliations:** ^1^ Basic and Clinical Translational Sciences, Tufts Graduate School of Biomedical Sciences, Tufts University School of Medicine, Boston, MA, United States; ^2^ Tufts Medical Center Rheumatology, Boston, MA, United States; ^3^ Department of Genetics, Molecular and Cell Biology, Tufts Graduate School of Biomedical Sciences, Tufts University School of Medicine, Boston, MA, United States

**Keywords:** CRISPR-Cas9, Sjögren’s disease, *TNFAIP3*, NF-κB, salivary gland, single-nucleotide polymorphisms, autoimmune diseases

## Abstract

Sjögren’s disease (SD) is a systemic autoimmune disease that particularly affects the salivary and lacrimal glands, causing sicca symptoms. Genetic polymorphism in the *TNFAIP3* gene has been implicated in the pathogenesis of SD. In this study, we aimed to functionally determine the impact of two specific single-nucleotide polymorphisms (SNPs) in *TNFAIP3*, rs6920220 (G/A) and rs2230926 (T/C/G), on the pathogenesis of SD. Using CRISPR-Cas9, we edited human salivary gland epithelial cells (SGECs) to incorporate *TNFAIP3* SNPs rs6920220 (G/A) and rs2230926 (T/C/G) and co-cultured them with Jurkat cells. We performed assays to examine gene expression, inflammatory cytokine levels, and related signaling pathways to investigate the effects of these genetic variants on *TNFAIP3* function and cellular response. Our results demonstrated that these SNPs reduced *TNFAIP3* expression, increased NF-κB activation, and elevated pro-inflammatory cytokine production. These findings provide strong evidence for the functional significance of these genetic variants in the pathogenesis of SD and underscore the utility of CRISPR-Cas9 technology in elucidating genetic contributions to autoimmune disorders.

## 1 Introduction

Sjögren’s disease (SD) is a systemic autoimmune disease that commonly manifests with dry eyes and dry mouth (known as sicca symptoms) (Sokhi et al., 2018). The common characteristics of the disease are chronic inflammation and functional tissue loss of salivary and lacrimal glands. Affected individuals can also develop extraglandular involvement in organs such as the joints, skin, lungs, gastrointestinal (GI) tract, nervous system, and kidney (Mariette and Criswell, 2018; Ramos-Casals et al., 2020). Clinical evaluation includes history and diagnostic tests to assess dryness of eyes and mouth, which includes the performance of a Schirmer test, slit-lamp exam with vital dye staining, and salivary flow rate evaluation of the salivary glandular function (Carsons and Patel, 2023). The most specific test is a minor salivary gland (lip) biopsy, which can demonstrate focal lymphocytic sialadenitis in positive specimens. Immunologically, SD is manifested by a higher level of autoantibodies against autoantigens SSA/Ro and SSB/La and hyperactivation of B and T lymphocytes (Sokhi et al., 2018). The disease pathophysiology includes underlying genetic and epigenetic susceptibility that leads to a dysregulated immune response including both innate and adaptive immune components (Shiboski et al., 2017; Mariette and Criswell, 2018; Bodewes et al., 2021). A chronic interferon signaling activity, altered frequencies of B- and T-cell subpopulations, and self-antigen-targeting autoantibody productions have also been reported (Yao et al., 2013; Sokhi et al., 2018; Lgiao et al., 2024).

Similar to any other autoimmune disease, genetic predisposition is one of the significant factors for developing SD. Population-based genetic studies have revealed the single-nucleotide polymorphisms (SNPs) and mutations that have functional relevance to the disease. Some studies reported the associations of HLA-TNF and non-HLA loci (IRF, STAT4, IL-12A, CXCR5, and TNIP1) (Ciccacci et al., 2019; Sokhi et al., 2018). Importantly, a candidate gene study by Musone et al. (2011) identified *TNFAIP3* and its related variants as a major contributing genetic position for SD-related pathogenesis. *TNFAIP3*, encoding the A20 protein, functionally suppresses NF-κB and TNF-induced apoptosis (Musone et al., 2011), which plays a critical role in preventing excessive inflammation and autoimmunity. Several genetic loci have been associated with increased susceptibility to SD, including the *TNFAIP3* gene (Ciccacci et al., 2019). Specifically, we focused on rs6920220 (G/A) as it has been consistently associated with increased SD risk in genetic studies and rs2230926 (T/C/G) as it is a nonsynonymous SNP that has been shown to alter A20 protein function.

The understanding of the cellular interaction between salivary gland epithelial cells (SGECs) and immune cells, such as B and T cells, can help reveal some critical aspects of the SD pathophysiology. Studies have demonstrated that SGECs can express co-stimulatory molecules such as CD80 and CD86, which are essential for T-cell activation and survival (Gauna et al., 2015). This suggests that SGECs are not merely passive targets of immune attacks but actively participate in shaping the immune landscape in the salivary glands. Furthermore, SGECs have been shown to induce B-lymphocyte survival and activation, even in the absence of direct antigen presentation, indicating a robust interaction that supports B-cell responses (Rivière et al., 2020). The infiltration of T cells, particularly CD4^+^ and CD8^+^ T cells, into the salivary glands is a defining feature of SD. Segawa et al. (2023) noted that in the context of sicca syndrome, there is a significant presence of CD3^+^ T cells in the salivary glands, which parallels the findings in SD in which T-cell infiltration is prominent. The infiltrated cytotoxic CD8^+^ T cells can induce apoptosis in SGECs through mechanisms such as granzyme-mediated cytotoxicity and the Fas/FasL pathway (Kaneko et al., 2022). This cytotoxic activity is further supported by evidence showing that activated CD8^+^ T cells can cause irreversible damage to ductal and acinar epithelial cells, leading to the characteristic secretory dysfunction observed in SD (Kaneko et al., 2022). The IL-6/STAT3 signaling pathway has been implicated in the expression of new autoantigens in SGECs, further linking the immune response to epithelial cell function and survival (Fujimura et al., 2017). This pathway not only promotes inflammation but also enhances the survival of infiltrating lymphocytes, creating a feedback loop that perpetuates the autoimmune process. The use of co-culture systems has allowed researchers to dissect the specific contributions of SGECs to T- and B-cell activation, providing insights into potential therapeutic targets for modulating the immune response in SD. In summary, the interaction between salivary gland epithelial cells and T and B cells in SD is characterized by a complex interplay of immune signaling, cellular activation, and tissue destruction.

In this study, using CRISPR-Cas9 gene editing, we generated SGEC lines carrying the *TNFAIP3* gene-associated risk alleles of rs6920220 (G/A) and rs2230926 (T/C/G) (Ray et al., 2020; Lee et al., 2022) to assess the impact of these SNPs on *TNFAIP3* expression and NF-κB activation in SGECs, examine the effect of these SNPs on pro-inflammatory cytokine and immune response gene expression in SGECs, and investigate how these SNPs modulate the interaction between SGECs and T cells in a co-culture system (Zhang et al., 2023).

## 2 Materials and methods

### 2.1 Cell culture

SGECs (cat. #T9289, Applied Biological Materials Inc.) were thawed and cultured in complete media (DMEM:F12 with 10% FBS and PSN) and maintained at 37°C in a humidified atmosphere with 5% CO_2_. The cells were passaged at approximately 80% confluency using 0.05% trypsin-EDTA. Jurkat cells (cat. #TIB-152) were maintained in RPMI-1640 with 10% FBS.

### 2.2 Cell co-culture

Transwell culture systems with cell inserts (pore size: 0.8 µm) were used to co-culture SGECs and Jurkat cells. Jurkat cells (1*10^6^ cells) were seeded on the upper chamber of the cell insert transwell, and SGECs (1*10^6^ cells) were seeded in the lower chamber of the transwell. This configuration physically separates the SGECs and Jurkat cells while allowing for the exchange of soluble factors, thereby simulating a paracrine interaction without direct cell-to-cell contact. The SGECs, which were seeded in the bottom chamber, were physically separated from the Jurkat cells, which were cultured in the upper chamber, and both were collected separately. Total RNA was then isolated independently from each distinct cell population (i.e., from the SGECs and the Jurkat cells) using standard RNA isolation protocols. RT-PCR was performed on purified cell-type-specific RNA samples and not on a mixed-cell RNA extract. This approach inherently ensured that the quantified mRNA signals were unequivocally derived from either SGECs or Jurkat cells, allowing us to accurately distinguish cell-type-specific transcriptional responses to the co-culture conditions. The co-cultures were kept as the control group and LPS (10 ng/mL)-treated group for SGECs with each risk alleles of rs6920220 (G/A) and rs2230926 (T/C/G) in replicates. LPS was used as an inflammatory inducer, which activates various immune pathways primarily through Toll-like receptor 4 (TLR4), expressed on many immune cells (Liu et al., 2023).

### 2.3 CRISPR-Cas9-mediated genome editing

To incorporate the desired SNPs into the genome of SGECs, homology-directed repair (HDR) using the CRISPR-Cas9 genome editing method has been used in this work. First, the donor Ultramer quality Alt-R HDR oligos were designed using the IDT online Alt-R HDR design tool.

The protocol involves co-transfection of gRNA (guide RNA) and HDR donor oligos (CRISPR-Cas9 reagents are stored at −20°C and always kept on ice while using). (HDR oligos and gRNA sequences are shown in Table1.)

**TABLE 1:** RT-qPCR primer list.

Sequence name	Sequence (5’---3′)
GAPDH forward primer	AGC​CAC​ATC​GCT​CAG​ACA​C
GAPDH reverse primer	GCC​CAA​TAC​GAC​CAA​ATC​C
IRF1 forward primer	GCA​CCA​GTG​ATC​TGT​ACA​AC
IRF1 reverse primer	GCT​CCT​CCT​TAC​AGC​TAA​AG
IRF5 forward primer	GGC​CTT​GGC​TTG​AAA​ACT​GG
IRF5 reverse primer	ATG​CGG​TCT​TTG​AGG​TCT​GG
NFKB2 forward primer	TCC​CAG​ATG​TAG​TGC​TGC​TG
NFKB2 reverse primer	AAG​GCC​AAT​ACG​TGG​TGA​AG
*TNFAIP3* forward primer	GCC​AAG​AGA​GAT​CAC​ACC​CC
*TNFAIP3* reverse primer	TTC​GTT​TTC​AGC​GCC​ACA​AG
IL-4 forward primer	GCC​TTC​AGC​ACA​TCT​TCA​CA
IL-4 reverse primer	ATC​ATC​GCT​TCT​CTG​CAC​CT
IL-6 forward primer	GGCTGCTCCTGGTGTTGC
IL-6 reverse primer	TCT​GAA​GAG​GTG​AGT​GGC​TGT​C
IL-8 forward primer	CAG​TTT​TGC​CAA​GAA​GTG​CTA​AAG
IL-8 reverse primer	GGG​TGG​AAA​GGT​TTG​GAG​TAT​GTC
IL-10 forward primer	GCC​CCT​GCT​CTC​ACC​TTA​AA
IL-10 reverse primer	GGG​CAT​CAA​AAA​GAC​CGC​AT
IL-12 forward primer	GGC​AGT​CTG​TGG​GGA​AGA​AC
IL-12 reverse primer	CAG​ATG​GCT​TGC​CTT​AGG​TCT
IL-13 forward primer	AGG​CAA​GCC​AAA​AGA​AGT​GA
IL-13 reverse primer	CCA​ACT​CTG​AGC​TCC​TGT​CC
IL-1β forward primer	GCA​GTC​TAC​ACA​GCT​TCG​GG
IL-1β reverse primer	CCG​CCT​CAG​CCT​CCC​AAA​G
PKR forward primer	TCT​TCA​TGT​ATG​TGA​CAC​TGC
PKR reverse primer	CACACAGTCAAGGTCCTT
OAS1 forward primer	TGC​ATC​AGG​AGG​TGG​AGT​TTG
OAS1 reverse primer	ATA​GAT​TCT​GGG​ATC​AGG​CTT​GC
CXCL10 forward primer	ATG​ACG​GGC​CAG​TGA​GAA​TG
CXCL10 reverse primer	TCA​ACA​CGT​GGG​CAG​GAT​AG

The oligos were resuspended in nuclease-free IDTE, and the guide RNA complex was prepared by combining crRNA and tracrRNA to a final concentration of 50 μM. The mixture was heated at 95°C for 5 min and cooled to room temperature (25°C). Next, the gRNA and Cas9 nuclease were combined to form a ribonucleoprotein (RNP) complex and incubated at room temperature for 15 min. Lipofectamine CRISPRMAX (cat. #CMAX00001) reagent was used to transfect cells in a 96-well plate. The RNP complex was mixed with HDR donor oligo and Cas9 Plus reagent (tube 1). CRISPRMAX reagent was diluted in media (tube 2). The solution in tube 1 was mixed into tube 2 and incubated for 15 min. The final solution was added to the cells and kept in an incubator for 72 h.

### 2.4 Genomic DNA isolation and probe-based real-time quantitative PCR

Genomic DNAs were isolated from SGEC lines using a Qiagen DNeasy kit (cat. #69506). To detect and quantify the desired SNP modifications by CRISPR-Cas9, primer- and probe-based real-time quantitative PCR was performed. PrimeTime Gene Expression Master Mix (cat. #1055770) from IDT was used. It is a 2X master mix solution containing a hot-start antibody, Taq polymerase, and other components needed for probe-based qPCR in two-step RT-PCR experiments with the reference dye included separately. In this 5′ nuclease-based assay, the primers and probe hybridize in a sequence-dependent manner to the complementary DNA (cDNA) strand. Because the probe is intact, the fluorophore and quencher are in proximity, and the fluorescence emitted from the fluorophore is absorbed by the quencher. The exonuclease activity of the polymerase cleaves the hybridized probe, and the fluorophore is separated from the quencher and fluoresces. These steps are repeated for each PCR cycle, which allow the detection of specific products. For the rs2230926 (T/C/G) SNP: for the T probe, Affinity Plus^®^ Mini Probe 5′6-FAM™/3′IB®FQ detected by the FAM/SYBR channel; for the C probe, Affinity Plus^®^ Mini Probe 5′SUN/3′IB®FQ detected by the VIC channel; and for the G probe, Affinity Plus^®^ Mini Probe 5′YAK^®^/3′IB®FQ detected by the ROX channel. For rs6920220 (G/A) SNP: for the WT probe, Affinity Plus^®^ Mini Probe 5′6-FAM™/3′IB®FQ detected by the FAM/SYBR channel and for the mutant (Mut) probe, Affinity Plus^®^ Mini Probe 5′YAK^®^/3′IB®FQ detected by the ROX channel. The probe sequences are provided in Table 1.

### 2.5 RNA extraction, cDNA preparation, and RT-PCR

Total RNA was extracted using a Quick-RNA Miniprep Kit (Zymo Research kit cat. #R1055) as per the manufacturer’s instructions. The extracted total RNA was quantified using the NanoDrop quantification system (Thermo Fisher Scientific). A measure of 500 ng of total RNA was converted to cDNA using an ABclonal ABScript Neo RT Master Mix synthesis kit (cat. #RK20433) according to the manufacturer’s instructions. Each real-time PCR used 1/20th of the cDNA, which was performed on the CFX96 Real-Time PCR system (Bio-Rad) using 2X Universal SYBR Green Fast qPCR mix (cat. #RK21203). The gene expression level was normalized with the housekeeping gene *GAPDH* as an endogenous reference and plotted as normalized expression using CFX96 Maestro software (Bio-Rad). Primers used for the real-time PCR analysis were as tabulated. The RT-PCR experiments were performed in replicates.

### 2.6 Statistical analysis

The data were analyzed using the t-test, and figures were constructed using GraphPad Prism. For statistical significance, p-values were determined and represented in graphs as * 0.01 < p < 0.05, ** 0.001 < p ≤ 0.01, *** 0.0001 < p ≤ 0.001, and **** p ≤ 0.0001. Each experiment was performed in replicates (n = 2) with technical triplicates (n = 3).

## 3 Results

### 3.1 *TNFAIP3* genetic variants in Sjögren’s syndrome and other autoimmune diseases

**FIGURE 1:**
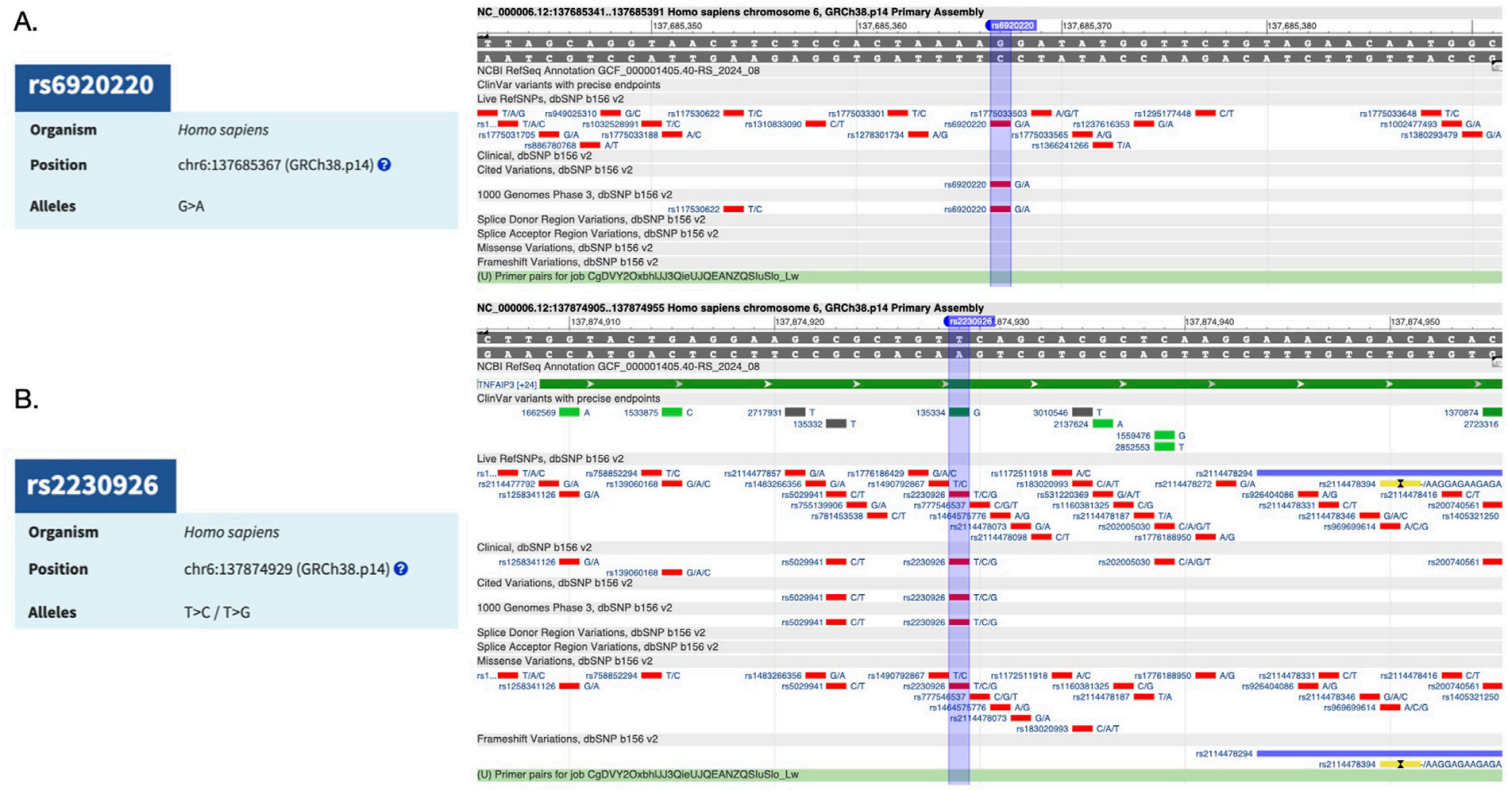
**(A)** *TNFAIP3*-associated risk factor SNP rs6920220 (G/A) and its reference sequence. **(B)** *TNFAIP3*-associated risk factor SNP rs2230926 (T/C/G) and its reference sequence.

The validation of SNPs associated with *TNFAIP3* as a risk factor through CRISPR-Cas9 technology offers a promising avenue for understanding the genetic underpinnings of a disease. Research has identified several SNPs within the *TNFAIP3* gene that are associated with autoimmune diseases. For instance, rs6920220 (G/A) has been linked to various autoimmune diseases, highlighting the importance of *TNFAIP3* in immune regulation (Wu et al., 2020; Ciccacci et al., 2019) (Figure 1A). Furthermore, other SNPs, such as the nonsynonymous SNP rs2230926 (T/C/G) (Figure 1B), have been shown to influence *TNFAIP3* expression and its inhibitory activity on NF-κB signaling (Odqvist et al., 2019; Ciccacci et al., 2019; Han et al., 2016). This SNP is significantly associated with increased susceptibility to autoimmune conditions, including systemic lupus erythematosus (SLE) and rheumatoid arthritis (RA) (Ciccacci et al., 2019; Das et al., 2018; Han et al., 2016).

### 3.2 CRISPR-Cas9 HDR mediated the introduction of *TNFAIP3* SNPs in human SGECs

To investigate the functional impact of specific genetic variants associated with SD, we utilized CRISPR-Cas9 HDR to introduce risk alleles into the *TNFAIP3* gene in human SGECs. We focused on the SNPs rs6920220 (G/A) and rs2230926 (T/C/G), which have been implicated in SD susceptibility.

For designing the single-nucleotide site-specific HDR donor templates and associated Cas9 guide RNAs, the IDT Alt-R HDR design tool was used. It is an easy-to-use, efficient online-based tool to design the oligonucleotides as donor templates and guide RNAs.

HDR donor templates were designed to introduce the desired SNP alleles (Table 1). For rs6920220 (G/A), the donor template (+) strand incorporated the A allele (ATC​TGC​TTC​CAT​CTG​TTA​GCA​GGT​AAC​TTC​TCC​ACT​AAA​AAG​ATA​TGG​TTC​TGT​AGA​ACA​ATG​GCA​TAT​GCA​GAC​AGT​GAT​C), whereas the (−) strand contained the T allele (GAT​CAC​TGT​CTG​CAT​ATG​CCA​TTG​TTC​TAC​AGA​ACC​ATA​TCT​TTT​TAG​TGG​AGA​AGT​TAC​CTG​CTA​ACA​GAT​GGA​AGC​AGA​T). The sgRNA sequence (ACT​TCT​CCA​CTA​AAA​GGA​TA) targeting the region surrounding rs6920220 (G/A) was selected for its predicted high on-target activity and minimal off-target potential.

For rs2230926 (T/C/G), the donor template (+) strand incorporated the G allele (GGC​GTT​CAG​GAC​ACA​GAC​TTG​GTA​CTG​AGG​AAG​GCG​CTG​TGC​AGC​ACG​CTC​AAG​GAA​ACA​GAC​ACA​CGC​AAC​TTT​AAA​TTC​CGC​TGG​CA), whereas the (−) strand contained the C allele (TGC​CAG​CGG​AAT​TTA​AAG​TTG​CGT​GTG​TCT​GTT​TCC​TTG​AGC​GTG​CTG​CAC​AGC​GCC​TTC​CTC​AGT​ACC​AAG​TCT​GTG​TCC​TGA​ACG​CC).

The sgRNA sequence (GGC​GCT​GTT​CAG​CAC​GCT​CA) targeting the region surrounding rs2230926 (T/C/G) was selected for its predicted high on-target activity and minimal off-target potential.

SGECs were transfected with Cas9 RNP complexes and the respective HDR donor templates. Control SGEC lines were also generated by SGEC transfection with a non-targeting control sgRNA using an Alt-R CRISPR-Cas9 Control Kit, Human, 2 nmol (cat. #1072554).

### 3.3 Allele-specific PCR confirms SNP editing

To confirm the successful introduction of the rs6920220 (G/A) and rs2230926 (T/C/G) SNPs in SGECs via CRISPR-Cas9 HDR, an allele-specific probe-based PCR was performed. The data demonstrate differential amplification across control and edited cell lines, indicative of successful gene editing. For desired SNP detection, we designed the IDT’s Affinity Plus SNP genotyping assay. This strategy is similar to the 5′ nuclease-based TaqMan assay. This assay employs two primers to amplify the sequence of interest containing the desired SNP, and two or three probes are designed to detect the SNP of interest. This strategy works on the competitive binding assay in which the probes compete to bind to the same sequence. The probe that perfectly complements the target sequence will outcompete other probes that mismatch the sequence. The probes are labeled with different fluorophores to detect the specific probe binding and SNP genotype detection (Table 1).

**FIGURE 2:**
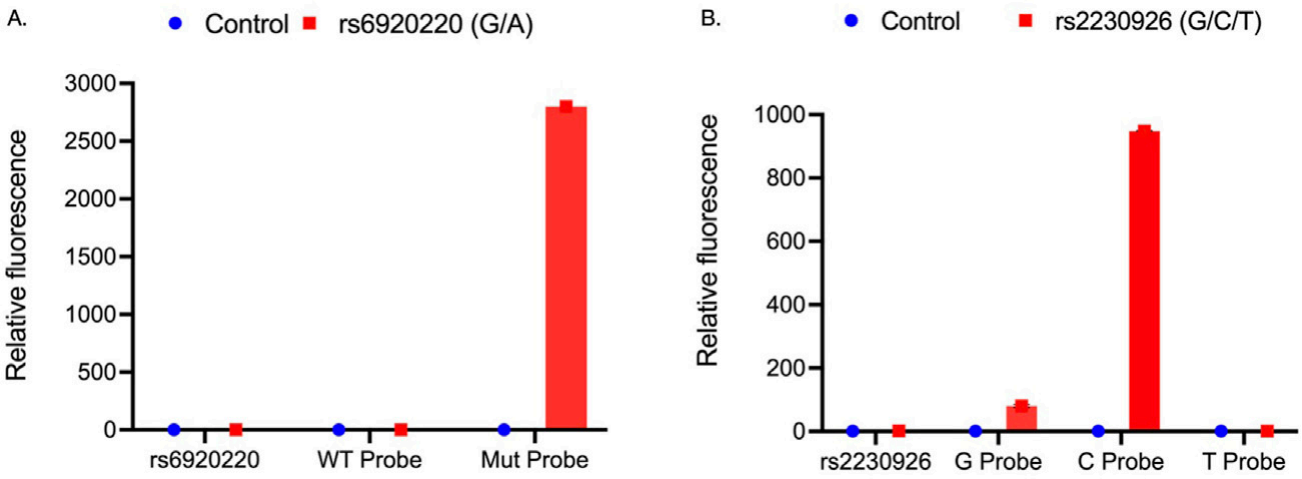
**(A)** Relative fluorescence of WT and Mut probes targeting the rs6920220 (G/A) site within the forward and reverse primer-amplified region of genomic DNA from CRISPR-Cas9-edited SGECs. **(B)** Relative fluorescence of G, C, and T probes at the rs2230926 (T/C/G) site within the corresponding PCR-amplified region of genomic DNA from edited SGECs. Each sample was analyzed in two independent runs, with three technical replicates per condition (n = 3 wells per group).

For rs6920220 (G/A), we used the abovementioned approach with specific probes for the A and G alleles. WT probe: 5′-TAAA + A + G + GA + TA + T + GGT-3’; Mut probe: 5′-TCT + C + CA + CTAAA + A + A + GA-3’; forward primer: 5′-TAC​GGC​AGC​GTA​ACA​TAG​TA-3′; and reverse primer: 5′-GTC​TGC​ATA​TGC​CAT​TGT​TCT​A-3′ to amplify the sequence of interest with an amplicon size of 125 bp. Similarly, for rs2230926 (T/C/G), probes were designed for the T probe: 5′-TG + CT + G + A + AC + AGCG-3’; C probe: 5′-TGCT + G + G + A + CAGC-3’; and G probe: 5′-TGCT + G + C + A + CA + GC-3′ with the forward primer: 5′-CAT​GCC​ACT​TCT​CAG​TAC​ATG-3′ and reverse primer: 5′-CGT​GTG​TCT​GTT​TCC​TTG​AG-3′ to amplify the sequence of interest with an amplicon size of 92 bp. The “+” symbol preceding a nucleotide in the probe sequence denotes synthetic modification of that nucleotide for better stability and hybridization efficiency. Probe-based RT-PCR analysis using genomic DNA from edited and control SGECs revealed that for the rs6920220 (G/A) variant, the Mut probe targeting the A allele showed a 2,799-fold increase in relative fluorescence compared to that in the control. In contrast, the WT probe targeting the G allele exhibited no significant difference in fluorescence relative to that in the control (Figure 2A).

Data for rs2230926 (T/C/G) demonstrated significant upregulation of the G probe (80-fold) and C probe (948-fold), whereas there is no significant upregulation of the T probe (Figure 2B). These data elaborated the presence of the targeted SNP alleles at the desired locations (Figure 2).

### 3.4 Functional validation of genome-edited SGECs and co-culture with Jurkat cells

**FIGURE 3:**
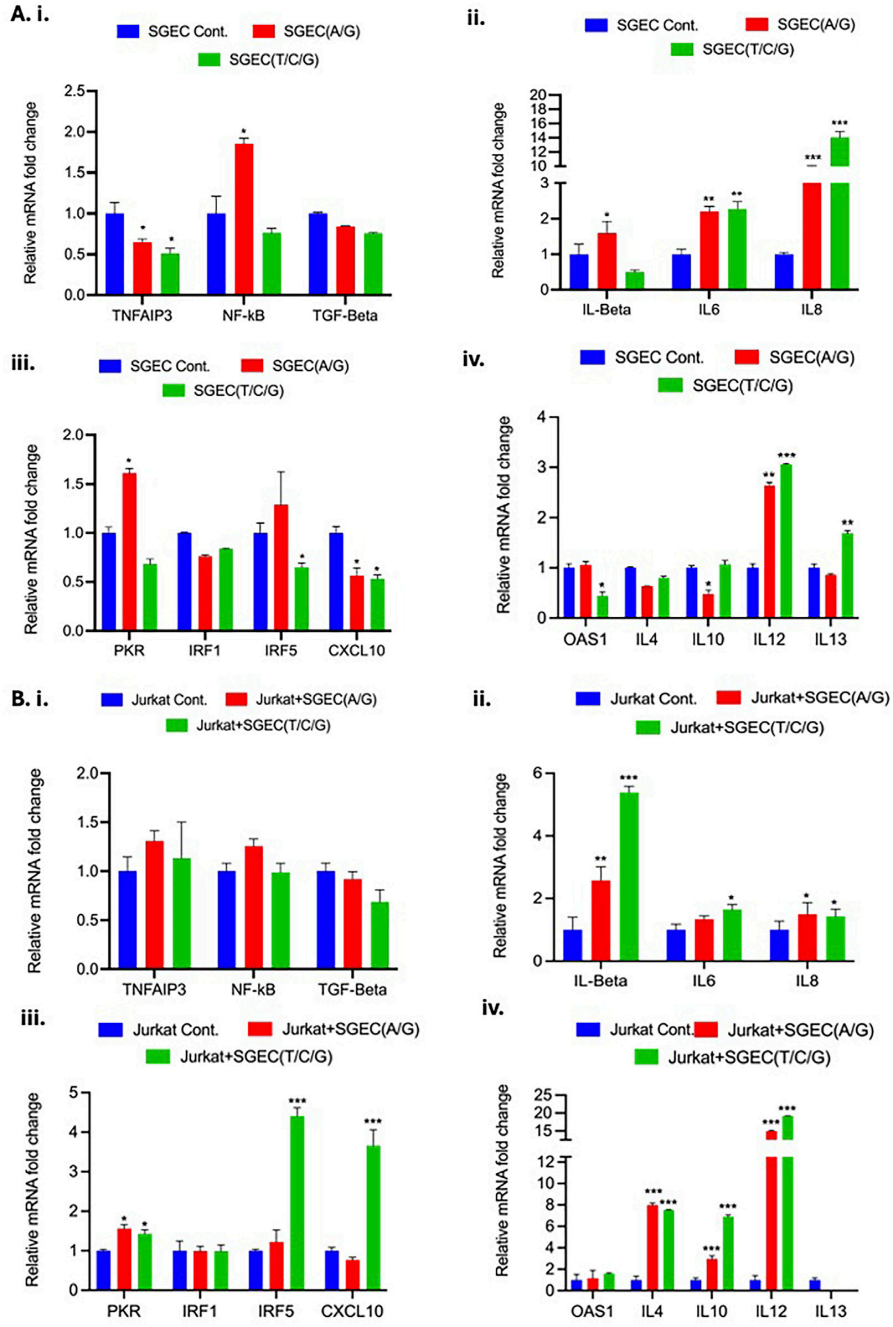
**(A)** i–iv, Relative mRNA fold changes in *TNFAIP3*, NF-κB, TGF-β, pro-inflammatory cytokines (IL-1β, IL-6, and IL-8), and immune-related genes (*PKR*, *IRF1*, *IRF5*, *CXCL10*, *OAS1*, *IL-4*, *IL-10*, *IL-12*, and *IL-13*) in SGECs with and without CRISPR editing. **(B)** i–iv, Corresponding expression levels in Jurkat cells co-cultured with SGECs. Gene expression was normalized to the housekeeping gene *GAPDH*. Data represent means ± SD from two biological replicates, each with three technical replicates (n = 3). Statistical significance was determined using Student’s t-test.

To investigate the functional impact of specific genetic variants associated with SD, we utilized CRISPR-Cas9 gene editing to generate SGEC lines carrying the risk alleles. We focused on variants within or near the genes implicated in SD pathogenesis, specifically examining the impact of rs6920220 (G/A) and rs2230926 (T/C/G). Following editing, we assessed the expression of key genes involved in inflammation and immune response in both SGECs and co-cultures of SGECs with Jurkat cells in a transwell setup, a model for immune cell interaction with the salivary gland epithelium. The cells were treated with LPS (10 ng/mL) in complete media to mimic and induce an inflammatory response. In SGECs, we observed a significant downregulation of *TNFAIP3* mRNA in both the rs6920220 (G/A) and rs2230926 (T/C/G) cells edited using CRISPR-Cas9 technology compared to the control SGECs (Figure 3Ai). This decrease was most pronounced in the rs6920220 (G/A) cells. NF-κB mRNA levels were markedly increased in rs6920220 (G/A) cells, indicative of increased NF-κB activity, a key driver of inflammation in SD. NF-κB mRNA expression in rs2230926 (T/C/G)-edited cells was not increased and instead showed a slight downward trend relative to that in the controls (Figure 3Ai). TGF-β expression, however, did not show significant changes across the groups.

Analysis of pro-inflammatory cytokines revealed a significant upregulation of IL-6 and IL-8 mRNA in both rs6920220 (G/A)- and rs2230926 (T/C/G)-edited SGECs (Figure 3Aii). IL-6 and IL-8 are known to be involved in pro-inflammatory and chronic inflammation signature as in the case of SD. IL-1β expression was also elevated, though not significantly. These data suggest that the presence of the risk alleles in SGECs leads to increased expression of pro-inflammatory cytokines, potentially contributing to the inflammatory milieu observed in SD. We also examined the expression of genes involved in immune responses, including *PKR*, *IRF1*, *IRF5*, and *CXCL10* (Figure 3Aiii). We observed a trend toward increased expression of IRF5 in rs2230926 (T/C/G)-edited cells, but this increase did not reach statistical significance (p = 0.754) (Figure 3Aiii). PKR is the serine/threonine kinase that plays an important role in innate immune response. IRF1 and IRF5 are the members of the interferon regulatory factor (IRF) family of transcription factors involved in immune response regulation. CXCL10 is the chemokine responsible for monocyte, natural killer cell, and T-cell migration. Further analysis focused on OAS1 and interleukins IL-4, IL-10, IL-12, and IL-13 (Figure 3Aiv). OAS1 is involved in innate cellular immune response. IL-4, IL-10, IL-12, and IL-13 are chemokines involved in pleiotropic effects such as immune response modulation, inflammation and related pathway, and B-cell and T-cell activity regulation. Similar to the previous set of genes, we observed some fluctuations, but none reached statistical significance. To mimic the interaction between SGECs and immune cells in the context of SD, we co-cultured the CRISPR-Cas9-edited SGECs with Jurkat cells. In the co-culture setting, we again observed a trend toward decreased *TNFAIP3* expression in the edited SGECs, although it did not reach statistical significance (Figure 3Bi). NF-κB expression showed a similar pattern of increase as seen in the SGEC monocultures without statistical significance. TGF-β expression remained unchanged. Co-culturing with Jurkat cells significantly impacted the expression of inflammatory cytokines. IL-1β, IL-6, and IL-8 were all markedly upregulated in the co-cultures with SGECs carrying the risk alleles, particularly rs6920220 (G/A) (Figure 3Bii). This suggests a synergistic effect of the genetic variants and immune cell interaction on inflammatory cytokine production. We also examined the expression of PKR, IRF1, IRF5, and CXCL10 in the co-culture setting (Figure 3Biii). Similar to the monocultures, IRF5 and CXCL10 showed a significant upregulation in rs2230926 (T/C/G) cells co-cultured with Jurkat cells. In the co-culture, we observed a significant increase in IL-12 mRNA levels in the presence of the rs6920220 (G/A)-edited SGECs (Figure 3Biv).

Other interleukins (IL-4, IL-10, and IL-13) showed some changes but were not statistically significant. These data demonstrate that the CRISPR-Cas9-mediated introduction of SD-associated risk alleles in SGECs impacts the expression of key inflammatory regulators. The presence of the A allele at rs6920220 (G/A) in particular and G and C alleles at rs2230926 (T/C/G) leads to decreased *TNFAIP3* expression and increased NF-κB activity, resulting in elevated pro-inflammatory cytokine production. Co-culturing with Jurkat cells further augmented this inflammatory response, highlighting the interplay between genetic predisposition and immune cell activation in the context of SD. These findings provide functional evidence supporting the role of these genetic variants in the pathogenesis of SD and suggest potential therapeutic targets for this debilitating autoimmune disease. All the RT-PCR primer sequences are listed in Table 2.

**TABLE 2:** CRISPR oligonucleotide sequences and probes.

Product or sequence name	Sequence
rs6920220 Alt-R HDR donor (+)	ATC​TGC​TTC​CAT​CTG​TTA​GCA​GGT​AAC​TTC​TCC​ACT​AAA​AAG​ATA​TGG​TTC​TGT​AGA​ACA​ATG​GCA​TAT​GCA​GAC​AGT​GAT​C
rs6920220 Alt-R HDR donor (−)	GAT​CAC​TGT​CTG​CAT​ATG​CCA​TTG​TTC​TAC​AGA​ACC​ATA​TCT​TTT​TAG​TGG​AGA​AGT​TAC​CTG​CTA​ACA​GAT​GGA​AGC​AGA​T
rs6920220 gRNA	ACT​TCT​CCA​CTA​AAA​GGA​TA
rs2230926 Alt-R HDR donor (+)	GGC​GTT​CAG​GAC​ACA​GAC​TTG​GTA​CTG​AGG​AAG​GCG​CTG​TGC​AGC​ACG​CTC​AAG​GAA​ACA​GAC​ACA​CGC​AAC​TTT​AAA​TTC​CGC​TGG​CA
rs2230926 Alt-R HDR donor (−)	TGC​CAG​CGG​AAT​TTA​AAG​TTG​CGT​GTG​TCT​GTT​TCC​TTG​AGC​GTG​CTG​CAC​AGC​GCC​TTC​CTC​AGT​ACC​AAG​TCT​GTG​TCC​TGA​ACG​CC
rs2230926 gRNA	GGC​GCT​GTT​CAG​CAC​GCT​CA
rs6920220 forward primer	TAC​GGC​AGC​GTA​ACA​TAG​TA
rs6920220 reverse primer	GTC​TGC​ATA​TGC​CAT​TGT​TCT​A
rs6920220 WT probe	TAAA + A + G + GA + TA + T + GGT
rs6920220 Mut probe	TCT + C + CA + CTAAA + A + A + GA
rs2230926 forward primer	CAT​GCC​ACT​TCT​CAG​TAC​ATG
rs2230926 reverse primer	CGT​GTG​TCT​GTT​TCC​TTG​AG
rs2230926 T probe	TG + CT + G + A + AC + AGCG
rs2230926 C probe	TGCT + G + G + A + CAGC
rs2230926 G probe	TGCT + G + C + A + CA + GC

## 4 Discussion and future perspectives

In this study, we used CRISPR-Cas9 gene editing to investigate the functional consequences of the *TNFAIP3* SNPs rs6920220 (G/A) and rs2230926 (T/C/G) in SGECs. Our results demonstrate that these SNPs lead to reduced *TNFAIP3* expression, increased NF-κB activation, and elevated pro-inflammatory cytokine production in SGECs, particularly in the presence of immune cells. Being a critical regulator of inflammatory NF-κB signaling, *TNFAIP3* has been strongly implicated in the pathogenesis of various autoimmune diseases, such as SLE and RA (Odqvist et al., 2019; Ciccacci et al., 2019; Han et al., 2016). This work mainly focused on exploring the functional impact of *TNFAIP3*-specific SNPs rs6920220 (G/A) and rs2230926 (T/C/G) in SD, which is characterized by systemic inflammation, lymphocytic infiltration, and dysfunction of the salivary and lacrimal glands. The successful introduction of SD-specific risk alleles rs6920220 (G/A) and rs2230926 (T/C/G) into SGECs provides a crucial *in vitro* model establishment for the disease. Through this study, we have also elaborated the establishment of isogenic cell lines differing only in the specific SNP. This allows for a more controlled study, omitting other potential interfering factors, to investigate the role of a particular SNP in disease consequences. However, limitations of this study include the use of Jurkat cells as a T-cell model, which may not fully replicate primary immune cell interactions. Although the SGECs utilized were not patient-derived and *TNFAIP3* SNP is associated with other rheumatic diseases, our study focuses on elucidating a fundamental molecular mechanism of *TNFAIP3*’s genetic variants in SGECs. For SNP rs2230926, T>C and T>G result in different amino acid substitutions. Thus, their effects on A20 function may differ; future studies are needed to investigate each variant individually. This mechanism of NF-κB-mediated inflammation is broadly implicated in the pathogenesis of both primary and secondary SD. Given that the interactions between SGECs and immune cells are central to the pathology of all forms of SD, our findings provide core mechanistic insights relevant to the broader disease continuum.

CRISPR-Cas9 editing of *TNFAIP3* variants in SGECs offers a powerful tool to investigate SD pathogenesis. However, this approach is primarily concerned with the off-target effect associated with CRISPR-Cas9 genome editing. The guide RNA, designed to target the specific locus, may exhibit partial complementarity to other sites in the genome, leading to unintended DNA modifications. The NGS-based methodologies for validation, such as whole-genome sequencing, are necessary to minimize these off-target events.

The rationale for the co-culture is to investigate how the presence of rs6920220 (G/A) and rs2230926 (T/C/G) risk alleles contributes to the interactions between SGECs and Jurkat cells to shape the inflammatory cascade related to SD. Generating this type of *in vitro* system allows for a comprehensive understanding of how genetic variants related to a key gene contribute to a disease phenotype. One limitation of our study is the use of the immortalized Jurkat cell line as a T-cell model. Although this allowed us to investigate the interaction between SGECs and T cells *in vitro*, Jurkat cells may not fully recapitulate the behavior of primary T cells from SD patients. Future studies should validate our findings using primary T cells or *in vivo* models. As this *in vitro* model is unable to completely represent the SD-associated tissue microenvironment and other systemic factors, findings from this type of studies need to be interpreted cautiously and validated in more complex *in vivo* models.

A deeper understanding of the functional consequences of *TNFAIP3* SNPs in SD has its own translational significance. Future research could explore additional *TNFAIP3* variants and their interplay with other factors in SD. This CRISPR-Cas9 model could also screen therapeutics targeting the *TNFAIP3*/NF-κB pathway, potentially leading to personalized treatments based on a patient’s *TNFAIP3* genotype. Ultimately, this type of approach will contribute to the development of more effective diagnostic and therapeutic approaches for similar debilitating autoimmune diseases.

## Data Availability

The original contributions presented in the study are included in the article/Supplementary Material, further inquiries can be directed to the corresponding authors.

## Supplementary material

SUPPLEMENTARY TABLE S1 Probe based PCR data analysis.

SUPPLEMENTARY TABLE S2 RT-qPCR data analysis of the related gene expression.
